# Factors associated with primary resistance to enfortumab vedotin in previously treated patients with metastatic urothelial carcinoma: a multicenter retrospective study

**DOI:** 10.1007/s10147-025-02822-1

**Published:** 2025-07-07

**Authors:** Daiki Ikarashi, Nozomi Hayakawa, Go Kaneko, Yuma Sakura, Yuki Endo, Ryo Yamashita, Suguru Shirotake, Yukihiro Kondo, Eiji Kikuchi, Wataru Obara

**Affiliations:** 1 Department of Urology, Iwate Medical University School of Medicine, Iwate, Japan; 2 Department of Urology, St. Marianna University School of Medicine, Kanagawa, Japan; 3 Department of Uro-Oncology, Saitama Medical University International Medical Center, Saitama, Japan; 4 Division of Urology, Shizuoka Cancer Center, Shizuoka, Japan; 5 Department of Urology, Nippon Medical School, Tokyo, Japan

**Keywords:** Early progressive disease, Enfortumab vedotin, Metastatic urothelial carcinoma, Primary resistance

## Abstract

**Background:**

To evaluate the primary resistance factors to enfortumab vedotin (EV) monotherapy by comparing treatment outcomes between the early progressive disease (EPD) group and non-EPD group.

**Methods:**

We retrospectively analyzed 121 patients with advanced urothelial carcinoma who received EV monotherapy across five institutions between 2019 and 2024. The patients were categorized into the EPD group (*n* = 34), defined by radiologically confirmed progressive disease within 3 months of EV initiation, and the non-EPD group (*n* = 87). The clinical parameters and oncological outcomes were compared between groups. The emergence of new metastatic lesions was defined as the detection of metastases in organs not previously identified as metastatic sites at baseline, during prior chemotherapy or immune checkpoint inhibitors (ICIs) before the initiation of EV.

**Results:**

The median overall survival was significantly shorter in the EPD group than in the non-EPD group (6.5 vs. 19.9 months, *p* < 0.001). The EPD group had a significantly higher incidence of new metastatic lesions and a lower prevalence of normal Hb levels. Multivariate analysis identified low Hb and the presence of new metastatic lesions as independent predictors of EPD. Among patients with new metastases in the EPD group, an average of 74% of lesions emerged during ICI treatment and 75% involved multiple foci. Notably, more than 50% of these new lesions showed progression at the same sites following EV therapy.

**Conclusions:**

Patients with low hemoglobin levels and new metastatic lesions before EV treatment may be at increased risk for EPD. For these patients, alternative treatment strategies should be considered before initiating EV.

**Supplementary Information:**

The online version contains supplementary material available at 10.1007/s10147-025-02822-1.

## Introduction

Urothelial carcinoma (UC) is among the most common malignancies, with bladder cancer accounting for approximately 95% of all UC cases [1]. Globally, bladder cancer was the ninth most frequently diagnosed cancer in 2022, with an estimated 614,000 new cases and 220,000 deaths reported [2]. Approximately 20% of patients are diagnosed with locally advanced or metastatic UC (la/mUC), necessitating systemic therapy such as platinum-based chemotherapy and immune checkpoint inhibitors (ICIs). However, most patients eventually develop disease progression and require additional lines of systemic treatment [3–5].

The advent of enfortumab vedotin (EV), an antibody–drug conjugate, has led to significant advances in the treatment of la/mUC following chemotherapy and ICI failure. EV has demonstrated promising clinical outcomes and has become a standard treatment option for previously treated UC. In the pivotal EV-301 trial, which formed the basis for regulatory approval, EV achieved a disease control rate of 71.9% and significantly improved overall survival (OS; median 12.88 vs. 8.97 months) and progression-free survival (PFS; median 5.55 vs. 3.71 months) compared with chemotherapy [6]. A Japanese subgroup analysis of the EV-301 trial also confirmed improved outcomes with EV in this population [7].

Despite its clinical benefits, a subset of patients’ experiences early progressive disease (EPD) shortly after the initiation of EV therapy, and the characteristics of these patients remain poorly understood. Additionally, while combination therapy with EV and pembrolizumab has recently been approved as a first-line treatment for treatment-naïve UC [8], monotherapy with EV still has clinical relevance. Such scenarios include disease recurrence following nivolumab plus gemcitabine–cisplatin therapy [9] or adjuvant nivolumab following neoadjuvant chemotherapy [10].

Erdafitinib, a fibroblast growth factor receptor (FGFR) inhibitor, has also emerged as a treatment option in this setting. The Phase III THOR trial demonstrated that erdafitinib significantly improved OS compared with chemotherapy in patients with FGFR alterations, including a Japanese cohort comparable with that in the EV-301 trial [11, 12]. As such, the choice between EV and erdafitinib for patients with ICI-refractory UC remains a matter of clinical consideration. Identifying predictors of EPD in patients treated with EV could provide valuable guidance in optimizing treatment selection.

In this multicenter retrospective study, we investigated factors associated with primary resistance to EV in patients with la/mUC to improve clinical decision-making in this patient population.

## Patients and methods

### Patient selection

**Fig. 1:**
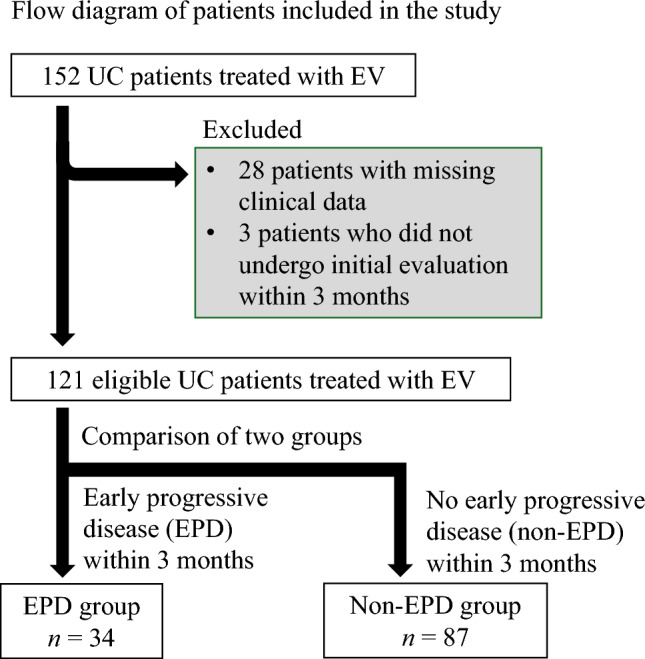
Flow diagram of patients included in the study. *EV* enfortumab vedotin, *UC* urothelial carcinoma

We retrospectively analyzed 152 patients with locally advanced or metastatic UC (la/mUC) who were treated with EV following prior chemotherapy and ICIs at five institutions between December 2021 and March 2024. A flowchart of the patient selection process is shown in Fig. 1. Twenty-eight patients with incomplete clinical data and three patients who did not undergo initial evaluation within 3 months of EV initiation were excluded. Ultimately, 121 eligible patients were included in the study.

The patients were stratified into two groups based on disease progression within 3 months of initiating EV: the EPD group (*n* = 34), defined as clinical or radiographic progression at first evaluation, and the non-EPD group (*n* = 87), defined as absence of progression within the same timeframe.

The baseline demographic and clinical characteristics—including age, sex, performance status, tumor histology, prior treatment history, metastatic sites, and laboratory values prior to EV administration—were collected from electronic medical records. The emergence of new metastatic lesions was defined as the detection of metastases in organs not previously identified as metastatic sites at baseline, during prior chemotherapy or ICIs before the initiation of EV. Chemotherapy and ICI treatment included neoadjuvant and adjuvant settings, and EV administration was performed for recurrence within one year after neoadjuvant and adjuvant therapy in this study. In patients who received neoadjuvant chemotherapy followed by postoperative adjuvant ICI, the baseline for identifying new metastatic lesions was defined as the initiation of neoadjuvant chemotherapy.

This study was approved through a centralized ethical review conducted by the Institutional Review Board of the St. Marianna University [Approval No. 6261 (B46)]. This centralized approval was subsequently approved by our institution (Approval No. C-IRB2023-045) and all other participating sites, and conducted in accordance with the Declaration of Helsinki.

### Treatment and response evaluation

As previously described [13], EV was administered at a dose of 1.25 mg/kg via intravenous infusion on Days 1, 8, and 15 of each 28-day cycle. The treatment decisions regarding the initial dose, interruptions, discontinuation, or dose reductions were made at the discretion of the attending physicians.

Although follow-up protocols varied by institution, the patients generally underwent computed tomography every 2–3 months. Tumor response was assessed according to the Response Evaluation Criteria in Solid Tumors (RECIST), version 1.1 [14]. Response categories included complete response (CR), partial response (PR), stable disease (SD), and progressive disease (PD). PD was also assigned to patients with clinical progression or cancer-related death as determined by the treating physician at the last follow-up. The disease control rate was defined as the proportion of patients achieving CR, PR, or SD. Adverse events were recorded and graded according to the Common Terminology Criteria for Adverse Events, version 5.0.

### Statistical analysis

Progression-free survival and OS were calculated from the date of the initial EV dose using the Kaplan–Meier method. Survival differences between the EPD and non-EPD groups were compared using the log-rank test. Clinicopathological variables between groups were analyzed using the Wilcoxon signed-rank test or analysis of variance, as appropriate.

Cox proportional hazards models were used for univariate and multivariate analyses to identify risk factors associated with EPD. The variables included in the multivariate analysis were selected based on previous studies investigating prognostic factors for OS in la/mUC patients [15, 16]. Continuous variables, such as hemoglobin (Hb) and C-reactive protein (CRP) levels, were dichotomized using median values as cutoffs. In addition, Hb was dichotomized using median values as cutoffs for male and female.

All statistical analyses were performed using JMP software (SAS Institute Inc., Cary, North Carolina). The *p* value of <0.05 was considered statistically significant.

## Results

The median patient age of EV initiation was 72 years (range: 29–92 years). The primary tumor site was the upper urinary tract in 65 (53.8%) patients, and 67 (55.4%) patients had undergone radical surgery prior to receiving EV. A total of 34 (28.1%) patients were classified in the EPD group. No patients in the EPD group discontinued EV due to adverse events or other reasons before progression was confirmed.

**Fig. 2:**
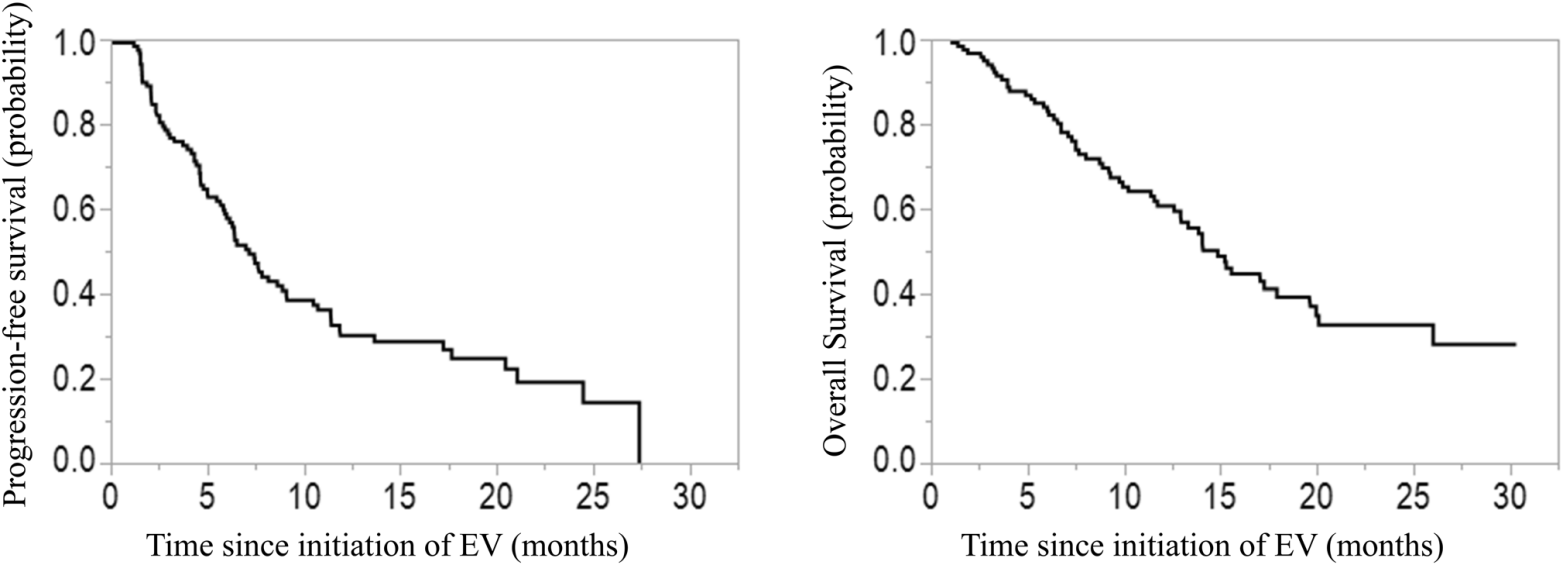
Kaplan–Meier curves of **a** PFS and **b** OS in the overall patient cohort treated with EV. *EV* enfortumab vedotin, *OS* overall survival, *PFS* progression-free survival

The median PFS and OS after EV initiation were 6.5 months (95% confidence interval [95% CI], 5.0–8.9 months) and 14.8 months (95% CI, 12.6–17.9 months), respectively (Fig. 2). The patients’ characteristics just before EV initiation of the EPD and non-EPD groups are compared in Tables 1 and 2. The EPD group had significantly lower Hb levels and a higher incidence of new metastatic lesions. Additionally, there were trends toward a higher frequency of bone metastases and elevated CRP levels in the EPD group. There were no significant differences in prior chemotherapy or ICI regimens between the groups.

**Table 1:** Patient characteristics before the initiation of EV

Variables	Overall (*n* = 121)	EPD vs. non-EPD		
		EPD (*n* = 34)	Non-EPD (*n* = 87)	*p*
Median age, year (range)	72 (29–92)	72 (29–85)	73 (46–92)	0.3014
Gender male, *n* (%)	84 (67.7%)	25 (73.5%)	59 (67.8%)	0.5398
ECOG PS, *n* (%)				
0	82 (67.7%)	23 (67.7%)	59 (67.8%)	0.5409
1	31 (25.7%)	10 (29.4%)	21 (24.1%)	
2	8 (6.6%)	1 (2.9%)	7 (8.1%)	
Primary tumor location, *n* (%)				
Bladder	56 (46.2%)	15 (44.1%)	41 (47.1%)	0.7654
Upper urinary tract	65 (53.8%)	19 (55.9%)	46 (52.9%)	
Periods from first-line treatment to the initiation of EV treatment, median (range) months	10.9 (1.3–50.6)	11.2 (1.3–37.8)	10.7 (1.6–50.6)	0.4513
Radical surgery (yes), *n* (%)	67 (55.4%)	18 (52.9%)	49 (56.3%)	0.7367
Histology, *n* (%)				
Pure urothelial carcinoma	89 (73.6%)	23 (67.7%)	66 (75.8%)	0.6108
Variant histology, *n* (%)	22 (18.2%)	8 (23.5%)	14 (16.1%)	
Others/unknown	10 (8.2%)	3 (8.8%)	7 (8.1%)	
Chemotherapy regimens				0.8904
GC/GCa	106 (87.6%)	29 (85.3%)	77 (88.5%)	
MVAC	6 (5%)	2 (5.9%)	4 (4.6%)	
Others	9 (7.4%)	3 (8.8%)	6 (6.9%)	
Immunotherapy regimens				0.6303
Pembrolizumab	80 (66.1%)	24 (70.6%)	56 (64.4%)	
Avelumab	29 (24%)	8 (23.5%)	21 (24.1%)	
Others	12 (9.9%)	2 (5.9%)	10 (11.5%)	
Total median tumor diameter before EV, mm (range)	49 (13–202)	59 (14–144.1)	45.5 (13–202)	0.4839
Site of metastasis before EV, *n* (%)^a^				
Lymph node only (without internal metastasis)	24 (19.8%)	6 (17.6%)	18 (20.7%)	0.706
Lung	58 (47.9%)	18 (50.0%)	40 (45.5%)	0.6903
Liver	21 (17.3%)	5 (13.9%)	16 (18.2%)	0.6878
Bone	31 (27.2%)	12 (36.1%)	19 (22.7%)	0.0844
Peritoneal dissemination	12 (9.9%)	3 (8.8%)	9 (10.2%)	0.7871
Local recurrence, *n* (%)	21 (17.3%)	9 (25.0%)	12 (13.6%)	0.1039
The number of patients with the emergence of new metastatic lesions before EV, *n* (%)	52 (42.9%)	22 (64.7%)	30 (34.5%)	0.0025

*ECOG PS* Eastern Cooperative Oncology Group performance status, *EPD* early progressive disease, *EV* enfortumab vedotin, *GC* gemcitabine + cisplatin, *GCa* gemcitabine + carboplatin, *MVAC* methotrexate + vinblastine + doxorubicin + cisplatin

^a^If multiple metastases were present, each site was counted separately

**Table 2:** Laboratory data just before the initiation of EV

Variables	Overall (*n* = 121)	EPD vs. non-EPD		
	Median (range)	EPD (*n* = 34)	Non-EPD (*n* = 87)	*p*
WBC	6400 (3200–28,520)	6370 (3200–28,300)	6395 (3300–28,520)	0.7864
Neutrophil	4192 (2851–26,780)	4331 (2380–25,158)	4040 (1851–12,731)	0.4262
Lymphocyte	1258 (224–5,217)	1332 (380–2516)	1177 (224–5217)	0.6159
Monocyte	428 (28–1470)	410 (186–935)	428 (149–1470)	0.6322
Hb				
Male	11.6 (7–15.4)	10.5 (7–13.6)	11.7 (7.9–15.4)	0.0182
Female	11.2 (7.4–14.3)			
PLT 10*3	251 (109–595)	269 (147–589)	248 (109–595)	0.125
LDH	200.5 (97–1296)	200 (97–1296)	200 (118–861)	0.6022
Alb	3.8 (1.6–4.6)	3.7 (1.6–4.5)	3.8 (2.3–4.6)	0.4297
CRP	1.04 (0.03–14.32)	2.27 (0.06–14.32)	0.62 (0.03–13.57)	0.0889
NLR at baseline	3.5 (0.7–42)	3.6 (1.03–17.78)	3.3 (0.71–42)	0.4195
LMR at baseline	2.9 (0.3–47.1)	2.9 (0.81–6.16)	3.0 (0.28–47.14)	0.5759
PLR at baseline	192.8 (49–2,151.7)	207.6 (84.9–647.6)	193.5 (49.1–2,151.7)	0.3384

*Alb* albumin, *CRP* C-reactive protein, *EPD* early progressive disease, *Hb* hemoglobin, *LDH* lactate dehydrogenase, *LMR* lymphocyte/monocyte ratio, *NLR* neutrophil/lymphocyte ratio, *PLT* platelet, *PLT/LMR* platelet/lymphocyte ratio, *WBC* white blood cell

**Fig. 3:**
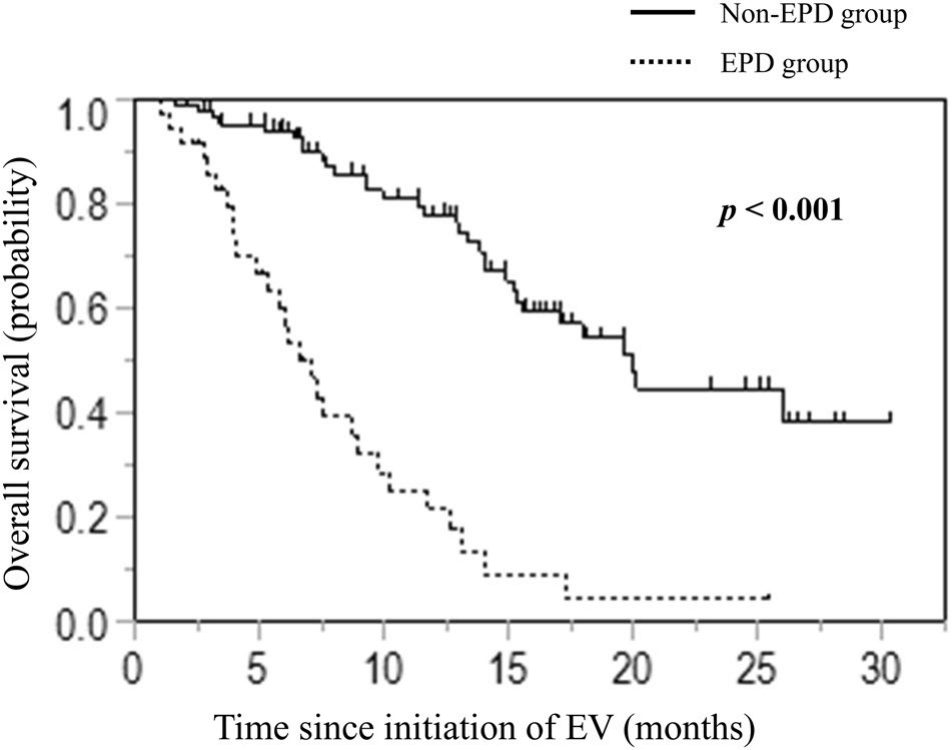
Kaplan–Meier curves of OS stratified by EPD and non-EPD groups treated with EV. *EPD* early progressive disease, *EV* enfortumab vedotin, *OS* overall survival

The median PFS was significantly shorter in the EPD group than in the non-EPD group (Supporting Information Figure S1: 1.7 vs. 10.7 months, *p* < 0.001). Similarly, the median OS was significantly shorter in the EPD group (Fig. 3: 6.5 vs. 19.9 months, *p* < 0.001).

The univariate and multivariate analyses of risk factors associated with EPD are shown in Table 3. In univariate Cox regression analysis, the presence of new metastatic lesions and low Hb levels were significantly associated with EPD. In multivariate analysis, both factors remained independent predictors of EPD: emergence of new metastatic lesions (hazard ratio [HR] = 2.71; *p* = 0.0105) and low Hb (HR = 3.34; *p* = 0.0067).

**Table 3:** Univariate and multivariate analyses for EPD

Variables	Early-progression disease					
	Univariate			Multivariate		
	HR	95% CI	*p*	HR	95% CI	*p*
ECOG PS, 1–2 vs. 0	1.15	0.56–2.37	0.689	1.16	0.51–2.64	0.7184
Site of metastasis before EV						
Liver, yes vs. no	0.83	0.32–2.16	0.7087	0.93	0.34–2.52	0.8912
Emergence of new metastatic lesions just before EV	2.56	1.27–5.19	0.0069	2.71	1.26–5.82	0.0105
Laboratory data median						
Hb, low vs. high	3.49	1.61–7.57	0.0015	3.34	1.39–7.99	0.0067
CRP, high vs. low	1.86	0.93–3.72	0.0793	1.03	0.43–2.18	0.9371

*CRP* c-relative protein, *ECOG PS* Eastern Cooperative Oncology Group performance status, *EPD* early progressive disease, *EV* enfortumab vedotin

Further analysis of new metastatic lesions in the EPD group is presented in Table 4. Among the 34 patients in the EPD group, 22 (64.7%) developed new metastatic lesions. The distribution of these new lesions—accounting for overlapping cases—was as follows: regional lymph nodes (*n* = 4), non-regional lymph nodes (*n* = 2), lungs (*n* = 8), liver (*n* = 4), bones (*n* = 8), and other sites (*n* = 2). Local recurrence was observed in six cases.

**Table 4:** Details of the new metastatic lesion in EPD group

Site of the new metastatic appearance	Total (*n*^a^)	Treatment timing of the appearance		Occurrence as multiple lesions (*n*, %)	The number of subsequent PDs at those new metastatic sites after EV (*n*, %)
		Chemotherapy (*n*, %)	Immunotherapy (*n*, %)		
Regional lymph node	4	2 (50%)	2 (50%)	4 (100%)	2 (50%)
Extra-regional lymph node	2	0	2 (100%)	1 (50%)	0
Lung	8	4 (50%)	4 (50%)	7 (87.5%)	6 (75%)
Liver	4	2 (50%)	2 (50%)	3 (75%)	2 (50%)
Bone	8	0	8 (100%)	5 (62.5%)	5 (62.5%)
Others	2	0	2 (100%)	–	1 (50%)
Local recurrence	6	2 (33.3%)	4 (66.7%)	–	3 (50%)

*EV* enfortumab vedotin, *PD* progressive disease

^a^Measurable for each organ, including duplicate cases

We also assessed the timing of new metastatic lesion development by treatment phase: 10 cases occurred during chemotherapy and 24 during ICI therapy. Most new lesions emerged as multiple foci rather than solitary lesions.

Furthermore, we evaluated whether the sites of new metastatic lesions during prior therapy were also sites of PD after EV treatment. The rates of subsequent progression at these new metastatic sites following EV were 50% for regional lymph nodes (2/4), 75% for lungs (6/8), 50% for liver (2/4), and 62.5% for bones (5/8).

## Discussion

This multicenter retrospective study demonstrated an association between EPD and poor prognosis in patients with locally advanced or metastatic UC (la/mUC) treated with EV after chemotherapy and ICIs. We identified two key predictors of EPD: the emergence of new metastatic lesions and low Hb levels. Notably, new metastatic lesions that developed before EV initiation were often refractory to subsequent EV treatment. To the best of our knowledge, this is the first report to focus on EPD in the context of EV therapy and to explore its predictive factors.

In real-world clinical practice, the observed median PFS and OS with EV treatment were 6.5 and 14.8 months, respectively. These values were slightly longer than those reported in the EV-301 trial [6]. Additionally, our results were comparable with outcomes reported in other Japanese prospective and retrospective EV studies [17–20], supporting the potential benefit of EV in real-world settings.

Low Hb is a component of the Bellmunt risk score, the only validated prognostic model for second-line therapy in UC [15, 16]. Therefore, the pretreatment Hb levels may serve as a simple yet valuable biomarker to identify patients who may benefit from EV.

We also found that the emergence of new metastatic lesions was significantly associated with EPD. Most new lesions developed during ICI therapy, rather than during chemotherapy. However, there were no significant differences in ICI regimens between the EPD and non-EPD groups, suggesting that the emergence of new metastatic lesions reflects treatment resistance regardless of the specific ICI agent used. Hara et al. [21] previously reported an association between the emergence of new lesions during pembrolizumab therapy and poor outcomes, possibly due to tumor heterogeneity. Based on these findings, we speculate that patients who develop new metastatic lesions during ICI therapy may follow an aggressive disease course in EPD group. On the other hand, the timing of new metastatic lesions in non-EPD groups also developed primarily during ICI therapy as well as EPD group. However, most of these new lesions appeared as solitary lesions rather than multiple foci. In addition, the incidence of subsequent progression at these sites after EV observed at the final evaluation was lower compared to EPD group (Supplemental Table 1). These differences suggest a distinct disease behavior and we hypothesize that underlying tumor heterogeneity may account for these results. These highlights the need to consider alternative treatment strategies, such as targeted therapy with erdafitinib, although its efficacy in patients with new metastatic lesions remains uncertain and warrants further investigation.

The emergence of new metastatic lesions may reflect resistance within the tumor microenvironment to both chemotherapy and ICIs. EV is an antibody–drug conjugate targeting Nectin-4, which is highly expressed in UC and has been proposed as a potential biomarker for EV efficacy [22, 23]. We hypothesize that new metastatic lesions may exhibit reduced or absent Nectin-4 expression, contributing to EPD. Previous studies have shown that Nectin-4 expression can decrease during metastatic evolution and may be absent in over one-third of patients with metastatic UC [24]. In our cohort, most new lesions progressed after EV administration within the same metastatic organ, supporting the possibility of Nectin-4 loss in these lesions. Nevertheless, further studies are needed to confirm the relationship between Nectin-4 expression and EV resistance.

It is also important to consider that EV resistance may result from mechanisms other than Nectin-4 downregulation [25]. Notably, in our study, the pre-existing lesions responded relatively well to EV, whereas newly developed lesions often did not, suggesting distinct resistance mechanisms. This supports the idea that tumor evolution leads to heterogeneity in treatment response.

Indeed, previous genomic analyses of metastatic UC have revealed substantial heterogeneity between primary tumors and metastatic lesions, indicating branched evolution during disease progression [26, 27]. Loriot et al. [27] reported that clinically actionable mutations—such as FGFR2/3 alterations (26%), PIK3CA mutations (13%), and ERBB2 and TSC1 mutations (7%)—were present across various metastatic sites with similar frequencies. These findings suggest that selecting targeted therapies based on molecular profiling may be a rational approach, particularly for patients at high risk of EPD.

Our study has several limitations. First, the retrospective design and limited sample size may have influenced the results. Nevertheless, our sample of 121 cases represents one of the largest cohorts to date and provides valuable real-world evidence from a multicenter setting. Second, due to its retrospective and multicenter design, imaging modalities and assessment intervals were not standardized across institutions. In particular, the use of unified imaging modalities, such as 18F-fluorodeoxyglucose positron emission tomography or enhanced CT, and the diagnostic criteria for liver and bone metastases varied, potentially introducing heterogeneity in metastatic evaluation. Therefore, a prospective study with standardized imaging protocols is needed to validate our findings in the future study. Third, Nectin-4 expression was not evaluated due to the practical challenges of obtaining biopsy specimens from multiple sites at specific treatment stages. Despite these limitations, our findings may inform future therapeutic strategies, including consideration of appropriate therapy for cases in which EPD is anticipated after EV. Although platinum rechallenge is considered an option, recent reports suggest that its efficacy after pembrolizumab or avelumab is limited when compared with EV [28, 29]. Thus, EV remains the preferred treatment in current clinical practice.

In conclusion, EPD is associated with poor outcomes in patients treated with EV. The presence of new metastatic lesions and low Hb levels before EV initiation are predictive of EPD. In such cases, individualized treatment strategies—including companion diagnostics and comprehensive genomic profiling—should be considered to optimize patient outcomes beyond EV therapy.

## Supplementary Information

Below is the link to the electronic supplementary material.

Supplementary file1 (DOCX 117 KB)

## Data Availability

The data supporting the findings of and the datasets generated and/or analyzed during this study are available from the corresponding author upon reasonable request.
