# Supplementary material for: Factors associated with primary resistance to enfortumab vedotin in previously treated patients with metastatic urothelial carcinoma: a multicenter retrospective study
Source: Int J Clin Oncol. 2025 Jul 7;30(9):1841–8. doi: 10.1007/s10147-025-02822-1 (PMC12378502; doi:10.1007/s10147-025-02822-1)
Supplement: Supplementary file 1 — Supplementary file1 (DOCX 117 KB) [file 10147_2025_2822_MOESM1_ESM.docx]

**Supplementary Information**

**Title:** Factors associated with primary resistance to enfortumab vedotin in previously treated patients with metastatic urothelial carcinoma: a multicenter retrospective study

**Journal name:** International Journal of Clinical Oncology

**Authors:** Daiki Ikarashi, Nozomi Hayakawa, Go Kaneko, Yuma Sakura, Yuki Endo, Ryo Yamashita, Suguru Shirotake, Yukihiro Kondo, Eiji Kikuchi, Wataru Obara

**Correspondence:** Daiki Ikarashi, MD, PhD

Department of Urology, Iwate Medical University School of Medicine, 2-1-1, Yahaba, Shiwa-gun, Iwate 028-3695, Japan.
Email: [dikara@iwate-med.ac.jp](mailto:dikara@iwate-med.ac.jp)


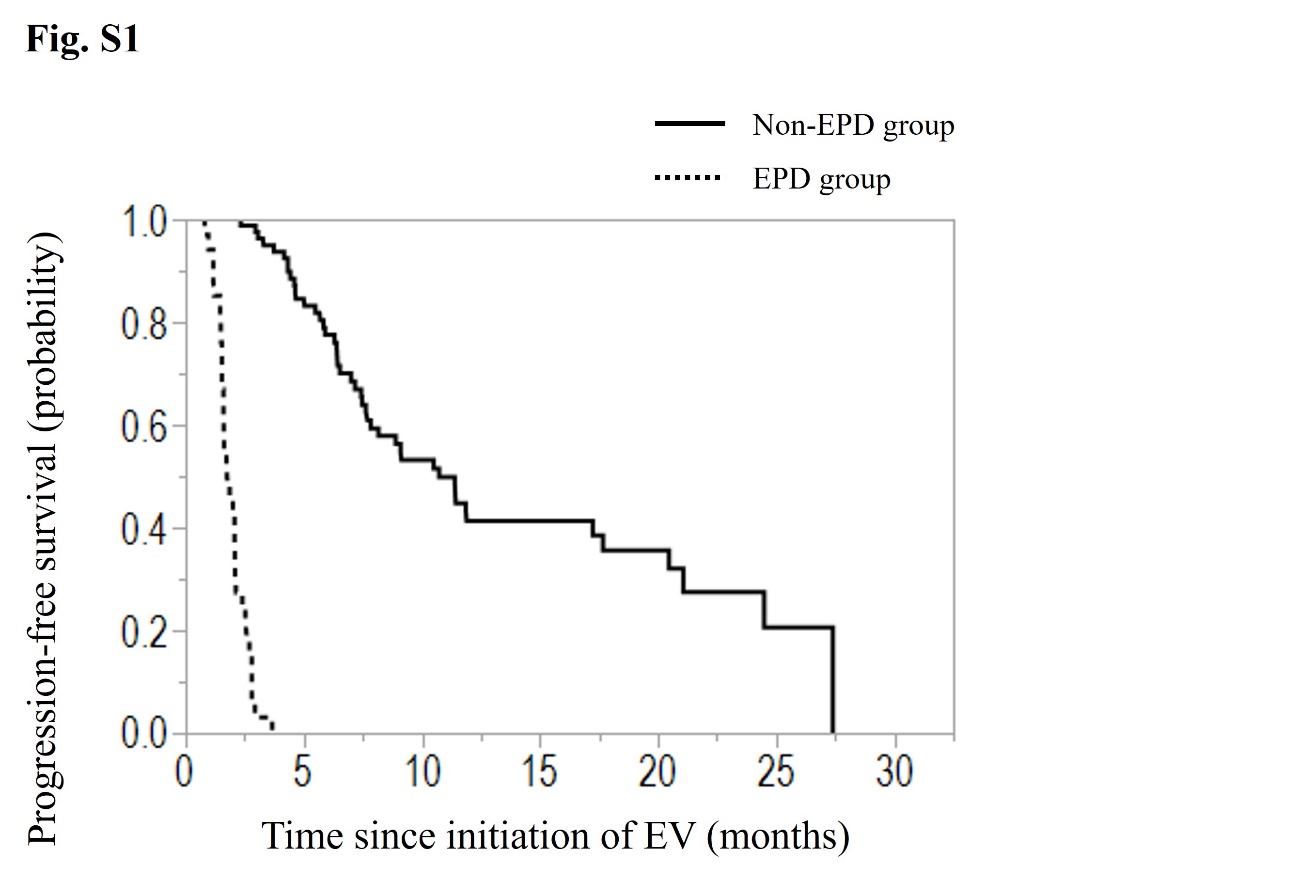


**Fig. S1** Kaplan–Meier curves of PFS stratified by EPD and non-EPD groups treated with EV

EPD, early progressive disease; EV, enfortumab vedotin; PFS, progression-free survival

**Supplemental Table 1.** Details of the new metastatic lesion in non-EPD group

| Site of new metastatic appearance | Total (n*) | Treatment timing of appearance | | Occurrence as multiple lesions (n, %) | the number of subsequent PD at those new metastatic sites after EV at final evaluation (n, %) |
| --- | --- | --- | --- | --- | --- |
|  |  | Chemotherapy   (n, %) | Immunotherapy (n, %) |  |  |
| Regional lymph node | 9 | 2 (22%) | 7 (78%) | 2 (22%) | 2 (22%) |
| Extra-regional lymph node | 3 | 0 | 3 (100%) | 1 (33%) | 0 |
| Lung | 9 | 4 (44%) | 5 (56%) | 3 (33%) | 5 (56%) |
| Liver | 5 | 1 (20%) | 4 (80%) | 2 (40%) | 1 (20%) |
| Bone | 8 | 2 (25%) | 6 (75%) | 5 (62.5%) | 2 (25%) |
| Others | 5 | 1 (20%) | 4 (80%) | - | 2 (50%) |
| local recurrence | 1 | 1 (100%) | 0 | - | 1 (100%) |
| PD; progressive disease, EV; enfortumab vedotin | | |  |  |  |
| * Measurable each organ including duplicate cases | | |  |  |  |
